# Estimation of an Elite Road Cyclist Performance in Different Positions Based on Numerical Simulations and Analytical Procedures

**DOI:** 10.3389/fbioe.2020.00538

**Published:** 2020-05-29

**Authors:** Pedro Forte, Daniel A. Marinho, Tiago M. Barbosa, Pedro Morouço, Jorge E. Morais

**Affiliations:** ^1^Department of Sports Sciences, Higher Institute of Educational Sciences of the Douro, Penafiel, Portugal; ^2^Department of Sport Sciences, Instituto Politécnico de Bragança, Bragança, Portugal; ^3^Research Centre in Sports, Health and Human Development (CIDESD), University of Beira Interior, Covilhã, Portugal; ^4^Department of Sport Sciences, University of Beira Interior, Covilhã, Portugal; ^5^Centre for the Study of Human Performance, Universidade de Lisboa, Lisbon, Portugal; ^6^Department of Sport Sciences, Polytechnic Institute of Leiria, Leiria, Portugal

**Keywords:** cycling, positions, analytical procedures, power, energy cost

## Abstract

The aim of this study was to use numerical simulations and analytical procedures to compare a cyclist's performance in three different cycling positions. An elite level road cyclist competing at a national level was recruited for this research. The bicycle was 7 kg and the cyclist 55 kg. A 3D scan was taken of the subject on the competition bicycle, wearing race gear and helmet in the upright position, in the handlebar drops (dropped position) and leaning on the elbows (elbows position). Numerical simulations by computer fluid dynamics in Fluent CFD code assessed the coefficient of drag at 11.11 m/s. Following that, a set of assumptions were employed to assess cycling performance from 1 to 22 m/s. Drag values ranged between 0.16 and 99.51 N across the different speeds and positions. The cyclist mechanical power in the elbows position differed from the upright position between 0 and 23% and from the dropped position from 0 to 21%. The cyclist's energy cost in the upright position differed 2 to 16% in comparison to the elbows position and the elbows position had less 2 to 14% energy cost in comparison to the dropped position. The estimated time of arrival was computed for a 220,000 m distance and it varied between 7,715.03 s (2 h:8 min:24 s) and 220,000 s (61 h:6 min:40 s) across the different speeds and positions. In the elbows position, is expected that a cyclist may improve the winning time up to 23% in comparison to he upright and dropped position across the studied speeds.

## Introduction

Competitive cycling is one of the most popular sports around the world. The different types of equipment, materials, designs, body positions, and training programs that are available can have an influence on the performance improvement by the bicycle-cyclist system. Thus, cycling biomechanics is an important topic for coaches, athletes, analysts, and sports scientists. Biomechanics and physiology are on the top of cycling practitioners' concerns (Minetti et al., 2001). The aerodynamics and efficiency of the bicycle-cyclist system are the main points of interest and concern in cycling biomechanics (Ettema and Lorås, 2009).

The cyclist's final race time depends on the acceleration (that by integrating it can yield the velocity). Thus, to reach a target speed, the propulsive forces (PF) must overcome the RF:

(1) $$\begin{array}{r} a=\;\frac{PF-RF}{m} \end{array}$$

where, a is the acceleration, PF the propulsive forces (applied by the cyclist's lower limbs on the crank), RF the resistive forces and m is the mass of the bicycle-cyclist system. Resistance is the sum of external forces in opposite direction (RF) of the bicycle-cyclist system motion. The drag (D) and rolling resistance (RR) are the external resistive forces (Candau et al., 1999) applied to the cyclist:

(2) $$\begin{array}{r} RF=D+RR \end{array}$$

In equation 1, RF is the drag and rolling resistance. Thus, to diminish the resistance cyclists can reduce D and RR or both. To reach the mean maximal velocity, cyclists may accelerate as soon as possible and keep the target speed as long as possible.

The energy cost plays an important role in the cyclist's physiology (Ettema and Lorås, 2009). The energy input must overcome the resistive forces at the target velocity (and thus, an estimated time of arrival):

(3) $$\begin{array}{r} v=\;\sqrt{\frac{2.E_{kin}}{m}} \end{array}$$

Where, E_kin_ e Ekin is the sum of the energy produced by the athlete–wheelchair system and the energy lost.

D is the main RF in cycling and is related with the cyclist positions and increases with speed (Gross et al., 1983; Kyle and Burke, 1984; Defraeye et al., 2010a; Debraux et al., 2011). The effective surface area (ACd) results from the multiplication of surface area (A) and coefficient of drag (Cd) (Forte et al., 2015, 2018b) and it is a mainstream procedure to assess cyclists' aerodynamics (Zdravkovic et al., 1996; Grappe et al., 1997; Candau et al., 1999; Defraeye et al., 2010a; Beaumont et al., 2018). Positions with smaller surface areas may also lead to less D. However, the body shape and equipment's design may affect the fluid flow and aerodynamics (Schlichting and Gersten, 2016). Moreover, the rolling resistance is possible to be minimized by choosing high-pressure tire, or light materials such as carbon and/or aluminum fibers to reduce the bicycle-cyclist system mass (Ryschon and Stray-Gundersen, 1993; Grappe et al., 1999).

Analytical procedures, experimental techniques (coasting down or wind tunnel testing) and numerical simulations enable one's to assess the RF (Debraux et al., 2011; Forte et al., 2015). Experimental techniques and numerical simulations are expensive, time consuming and required trained and dedicated researchers or technicians. Moreover, it is not possible assess or estimate cyclists performance in real-time (during a race). Conversely, analysis based on numerical simulations minimize confounding factors and control unpredictable environmental conditions (Forte et al., 2015). Experimental techniques are typically carried out over training and dedicated testing sessions or race events. Moreover, researchers and practitioners seek as much as possible to collect data in ecological settings, such as training sessions and competitions (Barbosa et al., 2017). To run analytical procedures it is required a set of assumptions (Forte et al., 2015; Barbosa et al., 2017). Based on those assumptions, it is possible to estimate with accuracy determinant outcomes such as the cyclist's mechanical power, energy cost and performance (Grappe et al., 1997; Martin et al., 1998; Forte et al., 2018a).

It is deemed as an efficient bicycle-cyclist system if one requires the minimal energy expenditure per unit of distance over time. Hence, cyclist should aim to expend the minimal amount of energy as possible at a specific speed (Lucia et al., 2003). Among the different strategies to improve the winning time and increase the efficient (thus, minimize the energy cost) cyclists use vary the body positions. It is possible to identify three main positions: (1) the upright position; (2) handlebar drops (dropped position) (3) leaning on the elbows (elbows position) (Defraeye et al., 2010a; Blocken et al., 2018). The numerical simulations seem to be one of the most precise methods to assess aerodynamics (Forte et al., 2015). RR dependents on the rolling coefficient and the mass of the system. Thus, a set of assumptions enables the assessment of the cyclist's performance. A range of studies have determined the influence of body position on the bicycle on the energy cost (García-López et al., 2008; Debraux et al., 2009; Fintelman et al., 2015). However, most of these studies have aimed at comparing time trial, standing/uphill cycling and upright positions. A few studies assessed the sprint position. Moreover, within peloton start at the beginning of the road race, cyclists are not able to use a time trial bicycle. Therefore, they try to lower the torso by placing their elbows on the handlebars. To date, there is limited research comparing the energy cost of this position with other cycling positions and as such examining the potential energy saving.

The aim of this study was to compare the cycling performance in three different positions based on numerical simulations and analytical procedures. It was hypothesized that the cyclist's performance may vary across the different positions and speeds. Moreover, positions with lowered torso (dropped and elbows position) might impose less drag in comparison to the upright position.

## Materials and Methods

### Participant

An elite level road cyclist participating at national level competitions was recruited for this research. The bicycle had 7.00 kg and the cyclist 55.00 kg of mass. All procedures were in accordance to the Helsinki Declaration regarding human research and a written consent by the volunteered subject was obtained beforehand.

### Scanning the Model

**Figure 1:**
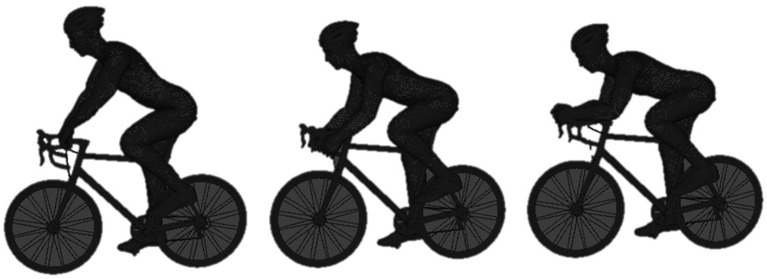
The meshed geometries in the three different positions: the upright position **(Left)**, dropped position **(Middle)** and time trial position **(Right)**.

The subject was scanned on his competition bicycle wearing racing gear and helmet. The scans were made in the upright, dropped and elbows position (Figure 1).

The scans were obtained by a Sense 3D scanner (3D Systems, Inc., Canada) and saved in the Sense Software (Sense, 3D Systems, Inc., Canada). The geometries were edited and converted to CAD models in Geomagic studio software (3D Systems, USA) (Forte et al., 2018b).

### Boundary Conditions

In Ansys Workbench software (Ansys Fluent 16.0, Ansys Inc., Pennsylvania, USA) was created a three-dimensional domain (length = 7 m; width = 2.5 m; height = 2.5 m) around the cyclist and meshed with more than 42 million of elements to represent the fluid. The cyclist geometry was at 2.5 meters of the inlet portion for each simulation (Blocken et al., 2013).

Typically, cyclist reach mean speeds about 11.11 m/s (~40 km/h) (El Helou et al., 2010). A speed of 11.11 m/s was set at the inlet portion of the enclosure (-z direction). The turbulence intensity was assumed as 1 × 10^−6^% for different positions. The surface of the bicycle-cyclist system was established as zero roughness non-slip wall and scalable wall functions were assigned.

### Numerical Simulations

The finite volume approach method allowed solving Reynolds-averaged Navier–Stokes (RANS) equations in Fluent CFD code (Ansys Fluent 16.0, Ansys Inc., Pennsylvania, USA). The Realizable k-e turbulence model was selected, and the SIMPLE algorithm used. The governing equations of the discretization schemes were defined as second and the gradients were computed by the least-squares cell-based method. Pressure and momentum were set as second order and second order upwind. The turbulent kinetic energy and dissipation rate were defined as first order upwind (Defraeye et al., 2010b). The convergence occurred automatically by the Ansys Fluent 16.0 (Ansys Fluent 16.0, Ansys Inc., Pennsylvania, USA).

### Outcomes

#### Drag Force

After the numerical simulations, it is possible to extract the coefficient of drag and surface area from Ansys Fluent Software (Ansys Fluent 16.0, Ansys Inc., Pennsylvania, USA). Then, the effective surface area (ACd) was computed. For the drag force, equation 4 was used:

(4) $$\begin{array}{r} F_{d}=\;\frac{1}{2}\rho AC_{d}v^{2} \end{array}$$

where, F_d_ is the drag force, C_d_ represents the drag coefficient, v the velocity, A the surface area and ρ is the air density (1.292 kg/m^3^). The C_d_ is given by re-arranging Equation 5:

(5) $$\begin{array}{r} C_{d}=\;\frac{\frac{1}{2}pAv^{2}}{F_{d}} \end{array}$$

#### Energy Cost

Computing drag and rolling resistance, equation 6 enables to assess the energy cost (i.e., energy expenditure per unit of distance) (Candau et al., 1999):

(6) $$\begin{array}{r} C=\;\frac{CR.m.g+\frac{\rho }{2}.A.C_{d}.v^{2}}{n} \end{array}$$

where, C is the energy cost, CR is the rolling coefficient, m the body mass of the bicycle-cyclist system, g the gravitational acceleration, v the mean velocity over the race, ρ the air density, A is the surface area and C_d_ the coefficient of drag and η the gross efficiency. The assumed gross efficiency of the cyclist was 20% (Bertucci et al., 2012) and CR 0.00368 (Candau et al., 1999). The differences between positions were presented with two ranges of speed: 1 to 11 m/s and 12 to 22 m/s. Upon that, the energy cost was estimated at the average speed of the Olympic Road Races and multistage tours (i.e., the Tour de France) winners, typically close to 40 km/h (≈ 11 m/s). As a result, the study has compared the energy cost between cycling positions near 30 km/h (≈ 8 m/s), 40 km/h (11 m/s) and 50 km/h (≈ 14 m/s). The selected road race distance to estimate the energy cost (in kilojoules, KJ) were 220 and 250 km.

#### Mechanical Power

Total mechanical power was estimated at speeds between 1 and 22 m/s (with increments of 1 m/s). The differences between positions were also assessed with two speed ranges: 1 to 11 m/s and, 12 to 22 m/s. The total net power (PNET, equation 12) was assessed by the sum of power to overcome drag (equation 7), power of bearing friction (PWB, equation 8), power of the rolling resistance (PRR, equation 9), Changes in Potential Energy (PPE, equation 10) and changes in kinetic energy (equation 11) (Martin et al., 1998):

(7) $$\begin{array}{r} P_{d}=F_{d}.v \end{array}$$

(8) $$\begin{array}{r} P_{WB}=v\;\left(91+87v\right)10^{-3} \end{array}$$

(9) $$\begin{array}{r} P_{RR}=CR.m.v.g \end{array}$$

(10) $$\begin{array}{r} P_{PE}=v.m.v.g \end{array}$$

(11) $$\begin{array}{r} P_{KE}=\frac{\Delta KE}{\Delta t}=\frac{1}{2}\left(m+\frac{I}{r^{2}}\right)\left(v_{f}-v_{i}\right)/\left(t_{i}-t_{f}\right) \end{array}$$

In equation 11, v_f_ is the final velocity, v_i_ the initial velocity, t_i_ the initial time and t_f_ the final time. KE is the additional kinetic energy stored in the rotating wheels (KE = $\frac{1}{2}$ I ω^2^). I is the moment of inertia of the two wheels (~0.14 kg.m^2^) and ω is the angular velocity of the wheels (proportional to bike and cyclist ground velocity given by: ω = v/r). Where r is the radius of the tire. Thus, kinetic energy stored in the wheels is given by KE = 1/2 I v^2^/r^2^.

(12) $$\begin{array}{r} P_{NET}=Pd+P_{WB}+P_{RR}+\;P_{PE}+\;P_{KE} \end{array}$$

(13) $$\begin{array}{r} P_{TOT}=\left(Pd+P_{WB}+P_{RR}+\;P_{PE}+\;P_{KE}\right)/E_{c} \end{array}$$

The total power (PTOT) can be computed by equations 12 and 13 where in equation 13, Ec is the chain efficiency factor, assumed as 0.976 (Martin et al., 1998). PTOT was estimated at each speed (1 to 22 m/s, with increments of 1 m/s) and position.

#### Performance

The estimated arrival time (ETA) at each mechanical power and speed was estimated by:

(14) $$\begin{array}{r} ETA=\frac{d}{v} \end{array}$$

computed by equations 8 and 9.

where v is:

(15) $$\begin{array}{r} v=\left(SUM\left(\frac{\sqrt{2.P_{TOT}}}{g*m*0.0053}+0.185\right)*60*\frac{60}{1000}*0.621\right)\\ \;*1.609 \end{array}$$

## Results

### Drag

**Figure 2:**
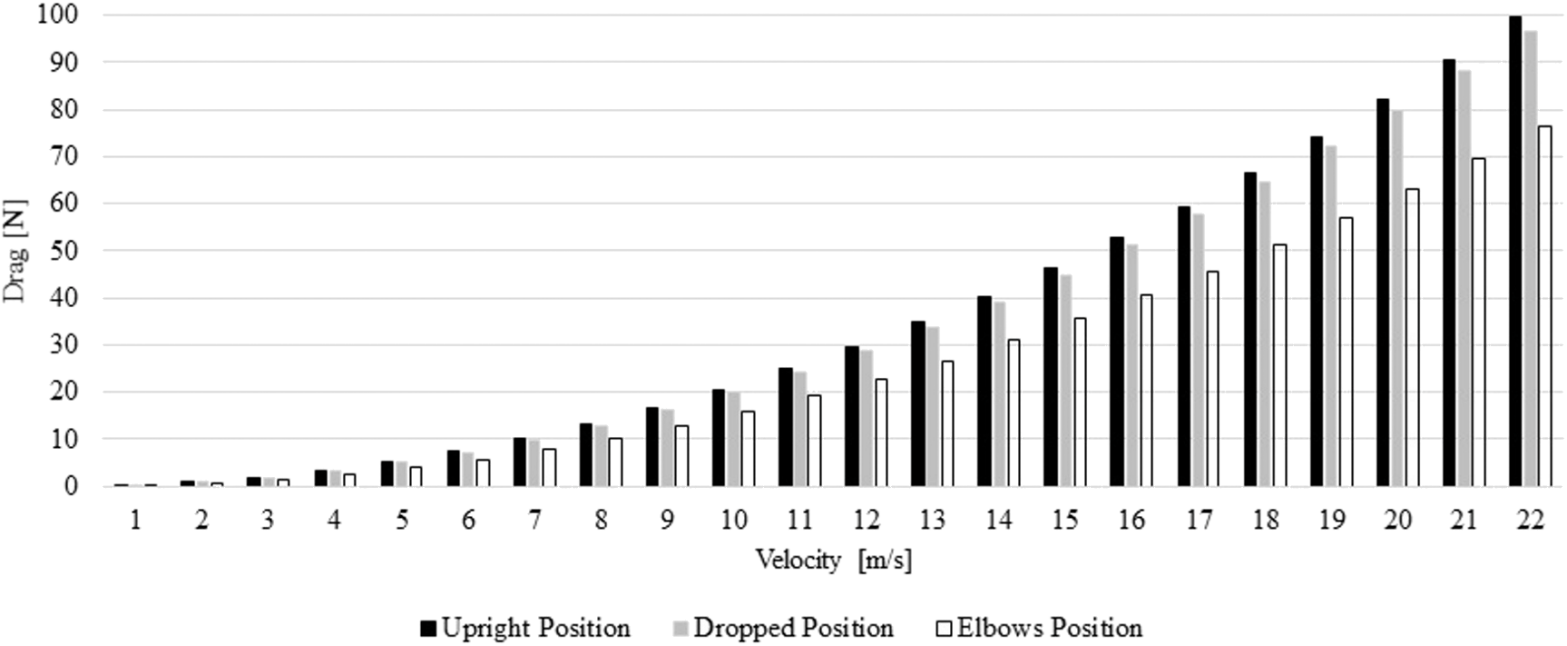
Drag force in the different positions and speeds.

The effective surface area was 0.332 m^2^ in the upright position, 0.327 m^2^ in the dropped position and 0.261 m^2^ in the elbows position. The drag values ranged between 0.16 and 99.51 N across the different speeds and positions (Figure 2). The elbows positions presented drag values between 0.16 and 76.45 N. The differences in percentage between the upright, dropped and elbows position are presented in Table 1. The elbows position imposed less drag in comparison to the upright and dropped positions across the different speeds.

**Table 1:** Percentage differences between the upright, dropped and elbows positions between 1 and 22 m/s.

**Positions**	**Dropped**	**Elbows**
Upright	3–5%	23–24%
Dropped		20–21%

### Mechanical Power

**Figure 3:**
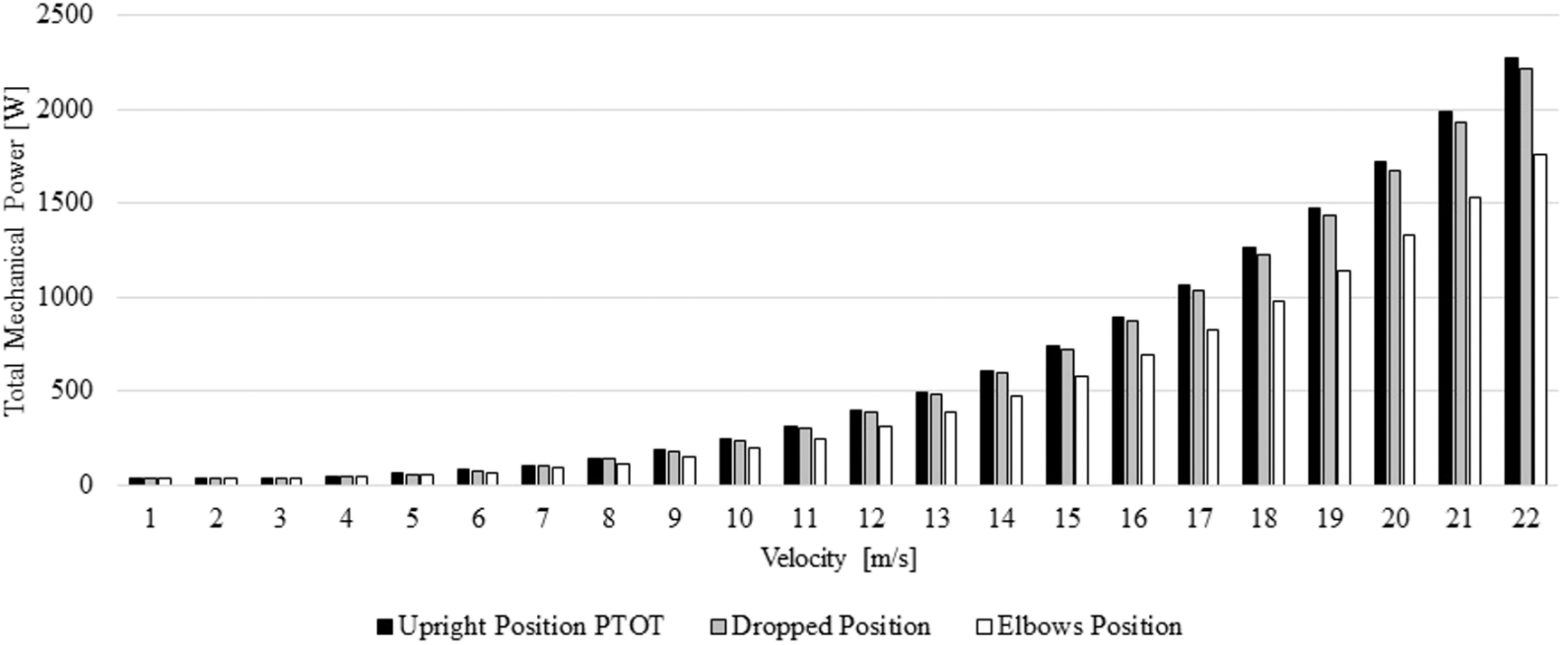
Total Power at different speeds and positions.

The cyclist mechanical power varied between 31.92 and 2274.65 W across different positions and speeds (Figure 3). The partial difference in mechanical power is depicted in Table 2. The elbows position required less mechanical power in comparison to the upright and dropped position across the different speeds.

**Table 2:** Mechanical power percentage differences from 1 to 11 m/s and from 12 to 22 m/s between the upright, dropped and elbows position.

**Velocity**	**1–11 m/s**
Positions	Dropped	Elbows
Upright	0.03–3%	0.16–21%
Dropped		0.13–19%
**Velocity**	**12–22 m/s**
Positions	Dropped	Elbows
Upright	3%	21–23%
Dropped		19–21%

### Energy Cost

**Figure 4:**
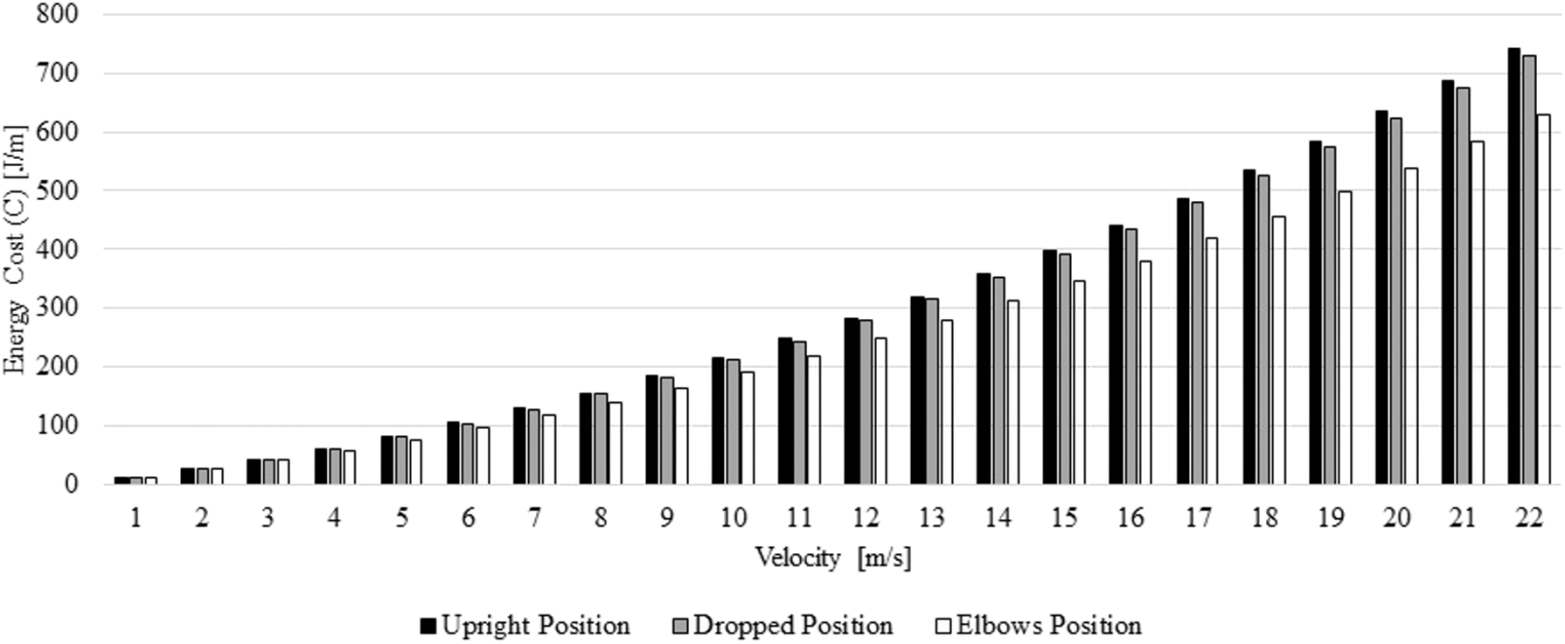
Energy cost at different speeds and positions.

In the selected positions and between 1 and 22 m/s the energy cost ranged between 11.98 and 743.51 J/m (Figure 4). The differences in percentage between positions and speeds are presented in Table 3. The elbows position required less energy cost, followed-up by the dropped and upright position, respectively.

**Table 3:** Energy cost percentage differences from 1 to 11 m/s and from 12 to 22 m/s between the upright, dropped, and elbows position.

**Velocity**	**1–11 m/s**
Positions	Dropped	Elbows
Upright	0.41–2%	2–13%
Dropped		2–10%
**Velocity**	**12–22 m/s**
Positions	Dropped	Elbows
Upright	2–13%	12–16%
Dropped		11–14%

**Figure 5:**
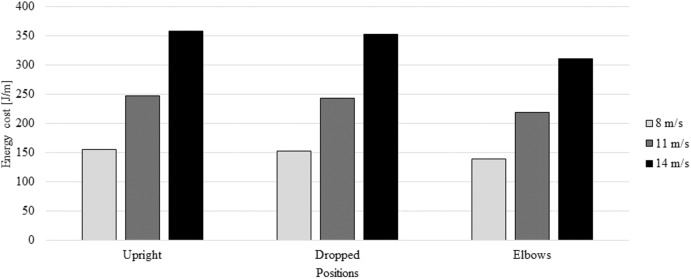
Energy cost (J/m) at 8, 11 and 14 m/s for upright, dropped and elbows positions.

At Olympic road events, the typical mean speed is 11 m/s (≈ 40 km/h). Thus, it is expected that the mean speed ranges between 8 m/s (≈ 30km/h) and 14 m/s (≈ 50km/h). Thus, the cyclist had an energy cost between 139.99 and 358.01 J/m across the different selected positions and speeds (Figure 5). The partial differences between positions at 4, 8, 11, and 14 m/s are presented in Table 4. Once more, the elbows position led to less energy cost followed-up, again by the dropped position and then the upright position.

**Table 4:** Energy cost percentage differences at 8, 11, and 14 m/s between the upright, dropped, and elbows position.

**Position (velocity)**	**Dropped (8 m/s)**	**Elbows (8m/s)**
Upright (8 m/s)	1%	10%
Dropped (8 m/s)		9%
**Position (velocity)**	**Dropped (11 m/s)**	**Elbows (11m/s)**
Upright (11 m/s)	1%	12%
Dropped (11 m/s)		10%
**Position (velocity)**	**Dropped (14 m/s)**	**Elbows (14m/s)**
Upright (14 m/s)	2%	13%
Dropped (14 m/s)		12%

**Figure 6:**
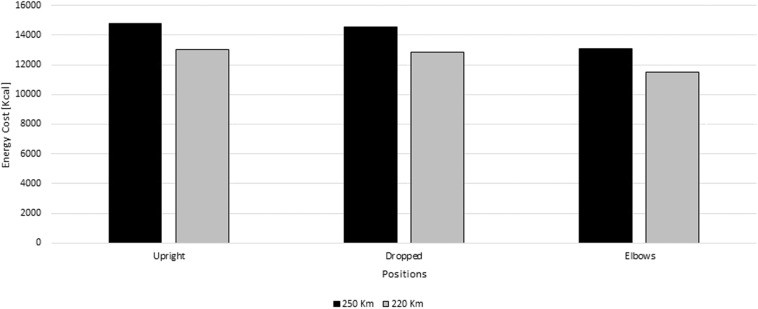
Energy cost (Kcal) for 250 km and 220 km race at 11 m/s for the upright, dropped and elbows positions.

The energy cost over an Olympic event (≈ 220–250 km) at 11 m/s ranged between 48076.21 and 61844.56 KJ (11490.49 and 14781.20 Kcal, respectively) (Figure 6). In the upright position, the energy cost ranged between 54423.21 KJ and 61844.56 KJ (13007.46 and 14781.20 Kcal, respectively). In the dropped position, it varied from 53653.21J to 60969.56 J (12823.43 and 14572.07 Kcal, respectively). In the elbows position, the cyclist's energy cost varied between 48076.21 and 54632.06 KJ (11490.49 and 13057.38 Kcal).

### Estimated Arrival Time (ETA)

**Figure 7:**
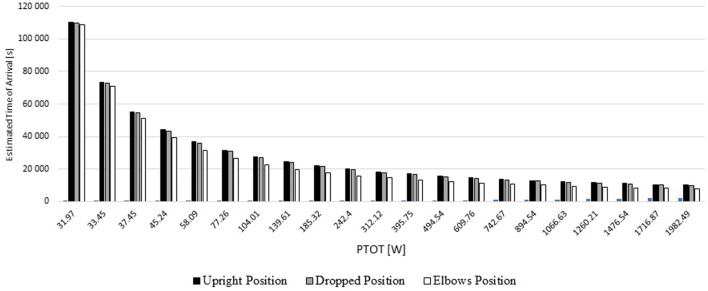
ETA for three different positions based on upright position PTOT.

The cyclist's ETA for a 220,000 m distance varied between 7,715.03 s (2 h:8 min:24 s) and 220,000 s (61 h:6 min:40 s) across the different speeds and positions (Figure 7). The position that required a longer ETA was the upright position followed-up by the dropped and then the elbows position. The ETA differences between the upright position and dropped position were 0.02–3.00%. The time gap between the upright position and elbows position was 0.15–23.00%. The dropped position differed from the elbows position between 0.13 and 23.00% across the different speeds.

At the mean speed of a road Olympic event (11 m/s ≈ 40 km/h), over 220,000 m (220 km), for a given mechanical power of 312.12 W, the cyclist may finish the race between 15836.98 and 20000 s (4 h:23 min:57.12 s and 5 h:33 min:36 s) across the different positions. In the upright position, the cyclist will take 20000 s (5 h:03 min:21.6 s. Whereas, in the dropped position the cyclist may finish the race in 19497.31 s (5 h:24 min:57.24 s) and in the elbows position 15836.98 s (4 h:23 min;21.6 s).

## Discussion

The aim of this study was to assess the performance of an elite cyclist in different positions and speeds by a set of analytical procedures. No research was found estimating the cyclists' performance and its determinants (i.e., drag, PTOT, C and ETA) in the three selected positions and at different speeds by a comprehensive set of analytical procedures based on numerical simulations. The main results were that: (1) drag, PTOT and C increased with speed; (2) the ETA diminished with speed and PTOT increased; (3) the elbows position presented lower mechanical power, energy cost and drag; (4) the cyclist may reach faster speed in the dropped and elbows position at the same mechanical power in the upright position; (5) the cyclist was able to deliver a better performance keeping-up in the elbows position.

Effective surface area (ACd) ranged between 0.261 and 0.332 m^2^ in the three positions at 11.11m/s (~40 km/h). In Olympic races, cyclists mean speed is near 40 km/h (11.11 m/s) (El Helou et al., 2010). It is possible to find in the literature ACd values between 0.261 and 0.42 m^2^ (Zdravkovic et al., 1996; Grappe et al., 1997; Candau et al., 1999; Defraeye et al., 2010a; Beaumont et al., 2018). It is expected that in the same conditions as in our study, drag reported in the literature varies between 0.16 and 131.32 N, in a range of speeds from 1 to 22 m/s. The drag values from the literature show good adherence to our study. In here, the drag values ranged between 0.16 and 99.51 N across the different speeds and positions.

The mechanical power ranged from 31.92 to 2274.65 W across the different positions and speeds. It is possible to find studies assessing the cycling mechanical power. González-Haro et al. (2007), noted a peak power of 355 W at 14.53 m/s in laboratory settings. In our study, at 15 m/s, the mechanical power ranged between 577.93 and 742.67 W. The differences can be explained by the settings where both studies where carried out (lab and velodrome vs. in-silicon settings) and the participants were triathletes. Conversely, Vogt et al. (2006) reported in professional road cyclists a power between 190 and 392 W at 11.41 m/s supporting the data of our study at 11 m/s. However, the Vogt et al. (2006) assessed the power with increment of 20 W each 3 min (started with 100 W). Grappe (2009), reported values of 250 W at a mean speed of 11 m/s in the time-trial position. Bogdanis et al. (1995) noted a peak power output of 1360 W during a 30 s sprint. These values are in accordance with our findings at speeds between 18 and 19 m/s, typically reached in sprinting events.

The differences between our results and literature can be explained by techniques selected to assess the power output. In our research total power was assessed by a set of analytical procedures; whereas, in the abovementioned studies power was measured in laboratory setting or with power meter devices in velodrome or stages road race. That said, overall the results in our study are in accordance to literature. The mechanical power is dependent of speed; thus, it is expected that mechanical power increases across the different speeds. The differences in mechanical power across positions were expected; hence, drag is minimized in the elbows position and mechanical power is also dependent of the resistive forces. Hubenig et al. (2011), assessed the power output in laboratory on a Velotron in trained females. The authors reported that elbows position showed less 4% of mechanical power in comparison to the upright position, supporting the differences between positions in our study.

Energy cost was computed based on the procedure reported by Candau et al. (1999). The gross efficiency was assumed to be 20% (Ettema and Lorås, 2009; Bertucci et al., 2012). Again, energy cost depends on drag, rolling resistance and gross efficiency (Candau et al., 1999). Thus, in road cycling, ACd variations may likewise increase or decrease the energy cost (Ryschon and Stray-Gundersen, 1993). In our study, the upright position had the highest energy cost, followed-up by the dropped position and then the elbows position. In a laboratory ergometer bicycle, the energy cost ranged between 1.11 and 2.39 J/m/kg (Belli and Hintzy, 2002). Thus, considering our subject an energy cost between 68.82 and 148.18 J/m was expected. Indeed, these values are in tandem to our results at speeds between 4 m/s and 8 m/s. In long distances (1500 km), the energy cost is near 25400 kJ (Saris et al., 1989). In our study, a race with 1500 km and mean speed of 11 m/s, yields an energy cost of 28500 kJ. These results again show good traction of our data.

Performance was assessed by estimated time of arrival (ETA). As far as our understanding goes, this is the first attempt to assess ETA based on mechanical power by a comprehensive set of analytical procedures. The cyclist's ETA was computed for 220,000 m and it varied between 7715.03 and 220,000 s across the different speeds and positions. The results of our study suggested that, a cyclist at the same mechanical power in the elbows position improves the wining time by 23% in comparison to the two other positions (upright and dropped position). That can be explained by: (1) the elbows position imposes less drag; (2) total power depends on drag and of drag is reduced, then so is the total power; (3) speed is dependent of the total power and the cyclist may reach faster speed in the dropped and elbows position at the same mechanical power in the upright position. Thus, for the same mechanical power, the elbows position improved more the winning time, followed-up by the dropped position.

Altogether, the position that imposes less drag, mechanical power, energy cost and ETA at speeds between 1 and 22 m/s was the elbows position, followed-up by the dropped and then the upright position. Positions with smaller surface area typically impose less drag (Grappe et al., 1997; Defraeye et al., 2010a). Thus, less drag leads to less total resistance, mechanical power and energy cost. This study brings awareness and insight to coaches, cyclists, sport scientists and researchers on the influence of the body position in road cycling performance. Coaches and other practitioners can use our data as reference to estimate and predict the mechanical power and energy cost of their cyclists over a race event. Cyclists should be aware that the elbows position minimizes drag and energy cost, leading to enhanced efficiency and improving the winning time.

It can be noted as limitations of this study that: (1) one single cyclist was assessed and the participant is just representative of the elite cyclists' cohort; (2) wearing different gear (e.g., helmet or clothing) may yield different results in the performance estimated; (3) a set of assumptions were used to estimate the resistive forces and total power.

## Conclusion

The drag, mechanical power energy cost and ETA of an elite cycling varies across different body positions and speeds. The elbows position imposes less drag, mechanical power, energy cost, and ETA followed by the dropped position and upright position. Cyclists may adopt the elbows position as much as possible during a race to become more efficient and thus improve the winning time.

## Data Availability Statement

The datasets generated for this study are available on request to the corresponding author.

## Ethics Statement

The studies involving human participants were carried out in accordance with the recommendations of the Declaration of Helsinki. The protocol was approved by the Technical and Scientific Committee of the Higher Institute of Educational Science of the Douro. The patients/participants provided their written informed consent to participate in this study.

## Author Contributions

PF, TB, and DM conceived and designed the experiments. JM and PF performed the experiments. PF, PM, and TB analyzed the data. PF and JM drafted the manuscript. PM, TB, and DM revised the manuscript.

## Conflict of Interest

The authors declare that the research was conducted in the absence of any commercial or financial relationships that could be construed as a potential conflict of interest.
